# Supplementary material for: Association between oral microbiome and seven types of cancers in East Asian population: a two-sample Mendelian randomization analysis
Source: Front Mol Biosci. 2023 Nov 21;10:1327893. doi: 10.3389/fmolb.2023.1327893 (PMC10702768; doi:10.3389/fmolb.2023.1327893)
Supplement: Supplementary file 1 [file DataSheet1.ZIP › Supplementary Materials/MR plots for tongue/tongue═╝/Pancreatic cancer/╥╚╧┘░⌐_╔╕╤í╜ß╣√/pheno.1497_to_pancreatic cancer_forest.pdf]

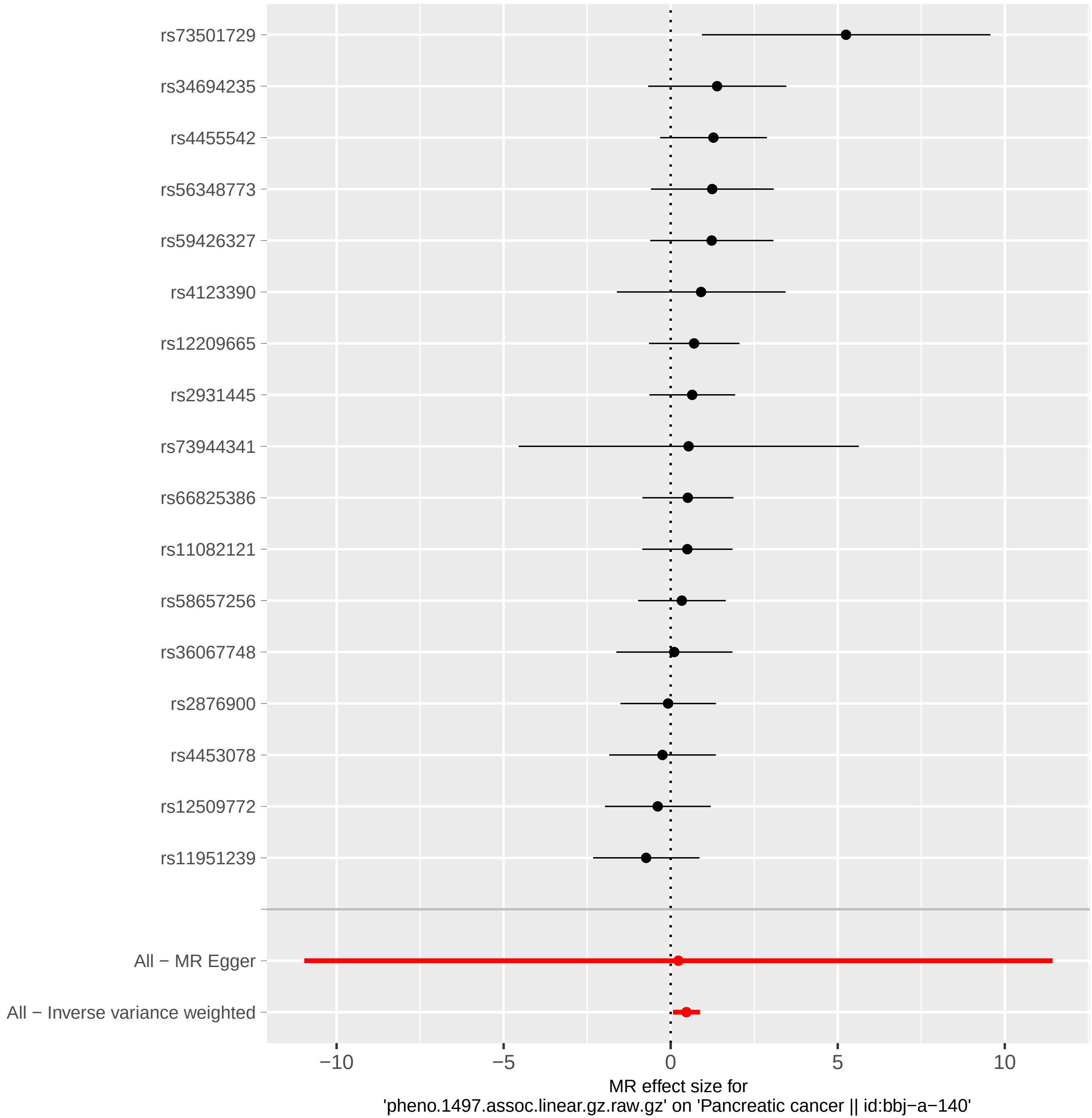

rs73501729
rs34694235
rs4455542
rs56348773
rs59426327
rs4123390
rs12209665
rs2931445
rs73944341
rs66825386
rs11082121
rs58657256
rs36067748
rs2876900
rs4453078
rs12509772
rs11951239
All − MR Egger
All − Inverse variance weighted
−10
−5
0
5
10
MR effect size for
'pheno.1497.assoc.linear.gz.raw.gz' on 'Pancreatic cancer || id:bbj−a−140'
